# Titanium Modulated the Occurrence States and Strain Aging Resistance of Residual Element Nitrogen in Scrap-Based Low-Alloy Steels

**DOI:** 10.3390/ma18214842

**Published:** 2025-10-23

**Authors:** Yuhe Huang, Haisheng Yang, Jun Lu, Jing Wang, Bicao Peng, Junheng Gao, Haitao Zhao, Honghui Wu, Chaolei Zhang, Shuize Wang, Xinping Mao

**Affiliations:** 1 Institute for Carbon Neutrality, University of Science and Technology Beijing, Beijing 100083, China; 2 Institute for Steel Sustainable Technology, Liaoning Academy of Materials, Shenyang 110004, China; 3 Hunan Iron and Steel Group Co., Ltd., Changsha 410100, China

**Keywords:** residual element, nitrogen, strain aging sensitivity, low-alloy steel, titanium microalloying, scrap-based steel

## Abstract

The steel industry is responsible for 7–9% of global CO_2_ emissions. Shifting from primary iron ore to recycled scrap in electric arc furnace (EAF) steelmaking offers significant decarbonization potential, reducing carbon intensity by 60–70%. However, increased scrap use in EAF operations leads to higher nitrogen absorption, which can degrade mechanical properties. Nitrogen dissolves into molten steel, where it forms Cottrell atmospheres at dislocations in the following processing steps, intensifying strain aging and reducing ductility. This study establishes a precipitation criterion based on the TiN solubility product to prevent harmful liquid TiN formation, enabling effective nitrogen fixation via fine TiN precipitates (5–20 nm). Multiscale characterization techniques, such as TEM and EBSD, show that Ti reduces the number of mobile N atoms by 60–70%, evidenced by a 50–65% decrease in Snoek/SKK peak intensities. Excessive titanium can refine ferrite grain size and prevents harmful TiN inclusions. Titanium microalloying presents a cost-effective, sustainable strategy to reduce strain aging in scrap-rich EAF steels, enabling more sustainable steel production without sacrificing material properties.

## 1. Introduction

Iron and steel manufacturing generates nearly one-tenth of global anthropogenic CO_2_ emissions, driving imperative decarbonization efforts. A cornerstone strategy involves substituting virgin iron ore with recycled ferrous scrap in metallurgical processes—each tonne of scrap utilization avoids approximately 1.5 tonnes of CO_2_ equivalent emissions [1]. This circular approach is optimally implemented through electric arc furnace (EAF) technology, where scrap constitutes more than 80% of charge materials. The EAF route demonstrates fundamentally superior carbon efficiency, exhibiting 60–70% lower greenhouse gas intensity relative to coal-intensive conventional blast furnace-converter (BF-BOF) pathways [2]. However, inherent metallurgical constraints impede broader EAF adoption, with nitrogen management constituting a primary technological challenge. In EAF operations, the intense electric arcs and high temperatures promote the dissociation of atmospheric N_2_ into highly reactive atomic nitrogen (N), which readily dissolves into molten steel [3]. Consequently, EAF steels often exhibit elevated levels of residual N, posing a major barrier to the production of high-quality, low-N steels.

Nitrogen’s influence on steel performance manifests as a state-dependent duality governed by concentration and microstructural interactions. At controlled levels, interstitial N enhances mechanical properties through solid-solution strengthening and precipitation hardening mechanisms [4]. Fine-scale nitride formation restricts grain boundary mobility during thermomechanical processing, refining microstructure concurrently. This principle is exploited in austenitic stainless systems where nitrogen alloying (0.1–0.25 wt%) stabilizes γ-phase crystallography, simultaneously enhancing pitting resistance and yield strength [5,6]. Conversely, excessive nitrogen dissolution—endemic to EAF processing with concentrations typically reaching 60–100 ppm—initiates detrimental phenomena including dislocation locking and intergranular embrittlement. These mechanisms fundamentally degrade mechanical integrity through strain aging susceptibility and cleavage fracture initiation.

These detrimental effects originate from nitrogen’s interstitial mobility and segregation kinetics. Solute N atoms preferentially diffuse to crystalline defects during cooling or deformation, forming Cottrell atmospheres that pin dislocations and creating brittle nitridic intergranular films (e.g., ε-Fe_2–3_N). While such segregation elevates yield strength through dislocation locking, it catastrophically compromises ductility and Charpy impact energy [7]. Furthermore, nitrogen supersaturation elevates the ductile–brittle transition temperature (DBTT) through dislocation immobilization mechanisms, exacerbating cold brittleness in service environments [8]. Additional failure modes include subsurface porosity from nitrogen repartitioning during solidification, macrosegregation-induced yield strength reduction, stress-concentrating TiN/AlN inclusions nucleating microcracks, and accelerated fatigue crack initiation at nitride–matrix interfaces [9,10]. The thermodynamic constraints of EAF processing render precise N management both technically challenging and economically prohibitive, frequently resulting in solute concentrations surpassing critical thresholds (typically > 60 ppm) [11,12]. Conventional mitigation approaches including vacuum processing and reactive gettering agents, incurring substantial operational costs that limit scalability for commodity steel production [11,12,13]. This challenge acutely impacts low-alloy structural grades exemplified by SAPH440, where the essential strength-toughness-weldability triad meets critical demand in automotive chassis components and structural frameworks. The scrap-intensive EAF route inherently elevates residual nitrogen to 60–100 ppm, intensifying dynamic strain aging phenomena during subsequent thermomechanical forming operations such as coil leveling. To reconcile decarbonization imperatives with stringent performance specifications, novel cost-effective approaches for modulating nitrogen’s occurrence state through microstructural engineering become indispensable.

Microalloying with strong nitride-forming elements presents a viable pathway for N management, where additions of niobium (Nb), vanadium (V), or titanium (Ti) immobilize interstitial nitrogen through precipitation of thermodynamically stable MN-type compounds. For instance, Nb and V are known to form fine carbonitrides that slow recrystallization and refine ferrite grains [14,15,16]. Titanium, due to its high affinity for nitrogen and lower cost compared to Nb and V, offers a promising alternative for nitride stabilization and strain aging suppression in scrap-based EAF steels [17,18,19].

Despite this potential, significant scientific uncertainties hinder optimal implementation. Critical among these are the undefined processing parameters required to avoid deleterious liquid TiN formation during solidification, unresolved relationships between Ti/N stoichiometry and nitrogen partitioning behavior, and competing microstructural consequences between strain aging suppression and precipitation evolution. This study systematically addresses these knowledge gaps by investigating Ti additions in scrap-based SAPH440 steel through integrated computational and experimental methodologies. Using integrated thermodynamic simulation, multiscale characterization, and mechanical testing, precipitation criteria were established to prevent harmful inclusions, quantify nitrogen’s transformation across solute/cluster/precipitate states, and elucidate titanium’s mechanistic role in enhancing strain aging resistance while optimizing strength-ductility synergy. The scientific novelty of this work lies in the establishment of a TiN precipitation criterion for scrap-based EAF steels, coupled with multiscale evidence of Ti-mediated nitrogen immobilization and strain aging suppression, providing a cost-effective pathway for high-performance, low-carbon steel production.

## 2. Materials and Methods

The experimental material in this study was commercial-grade SAPH440 low-alloy steel produced via the scrap-based electric arc furnace (EAF) process. In conventional converter steelmaking, the N content in the final molten steel typically stabilizes at approximately 40 ppm. To systematically investigate the role of Ti in regulating N behavior, a series of samples with controlled Ti/N ratios were designed. Although Ti effectively fixes N by forming stable compounds, its high affinity for N, sulfur (S), and oxygen (O) may inadvertently promote inclusion formation. Notably, TiN precipitates formed in the liquid phase are particularly detrimental due to their coarse particle size (>1 μm) and sharp cubic morphology, which act as stress concentrators and markedly degrade material ductility. To suppress the formation of these harmful precipitates, the solubility product of liquid TiN was adopted as the key design criterion for alloy composition optimization [14,15,16]:

lg([%Ti][%N])_L_ = 5.9 − 16586/T (1)

lg([%Ti][%N])_δ_ = 5.56 − 17205/T (2)

**Figure 1:**
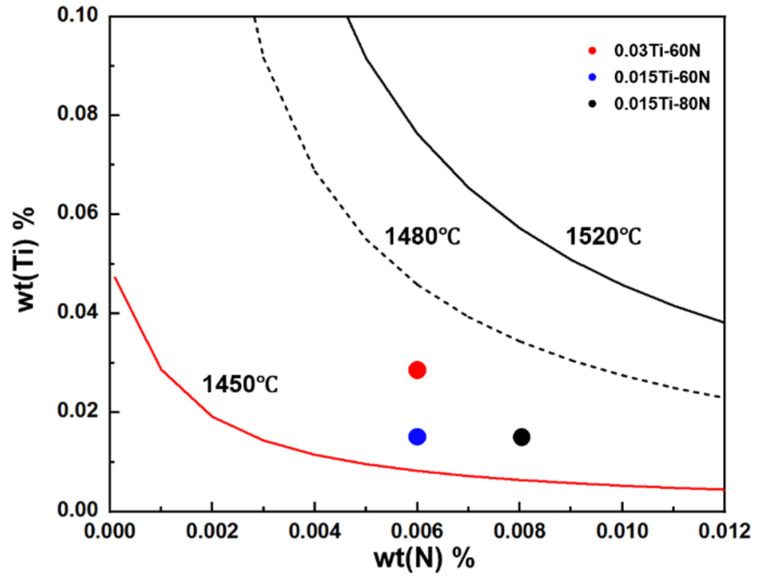
The solubility product curve of TiN in liquid and solid steel.

Using Equations (1) and (2), where [%Ti] and [%N] represent the mass percentages of titanium and nitrogen in the steel, respectively, and T is the absolute temperature in Kelvin (K), the critical concentrations of Ti and N required to prevent the formation of liquid TiN were determined (Figure 1). The solid curves represent the calculated solubility products of TiN at different temperatures (1450 °C, 1480 °C, and 1520 °C). The 1520 °C curve corresponds to the liquid state condition. As shown in Figure 1, for N levels below 100 ppm, Ti additions of ≤0.03 wt.% effectively prevent liquid TiN formation, allowing the N to precipitate in the solid phase. Based on this criterion, four experimental steels with controlled Ti/N ratios were designed: 80N (reference, with 80 ppm N), 0.03Ti-60N (with 0.03 wt.% Ti and 60 ppm N), 0.015Ti-60N (with 0.015 wt.% Ti and 60 ppm N), and 0.015Ti-80N (with 0.015 wt.% Ti and 80 ppm N). Additionally, to ensure the steel’s strength remains stable after Ti addition, the Mn content in the Ti microalloyed samples was reduced from 1.4 to 1 wt.%. The chemical compositions of these experimental steels are presented in Table 1.

The investigated material was subjected to industrially relevant thermo-mechanical processing through laboratory hot-rolling simulation. Initial homogenization conducted at 1200 °C for 60 min to ensure chemical uniformity. Subsequent hot-rolling comprised five consecutive deformation passes in the temperature range of 1050 °C to 830 °C, with a thickness reduction from 40 mm to 3 mm (approx. 25% reduction per pass). Finish rolling was conducted at 830 °C, followed by accelerated cooling to 550 °C and simulated coiling. The chemical composition of the experimental material was determined by wet chemical analysis. The measured composition is presented in Table 1. The critical phase transformation temperatures were determined using thermodynamic calculations performed with the Thermo-Calc software and the TCFE9 database. For microstructural characterization, longitudinal sections were mechanically prepared through sequential grinding and diamond polishing, with final microstructural revelation using 2% nital etching. Comprehensive analysis employed a scanning electron microscopy (TESCAN Mira LMS, TESCAN, Brno, Czech Republic) equipped with a secondary electron (SE) detector, coupled with electron backscatter diffraction (Oxford Symmetry S2, Oxford Instruments, Oxford, UK) at a 0.2 μm step resolution, utilizing specimens prepared by electrochemical polishing in 5% perchloric acid/ethanol solution. The grain sizes were measured from SEM images using the linear intercept method according to the ASTM E112 standard. Complementary transmission electron microscopy (JEOL JEM 200F, JEOL Ltd., Tokyo, Japan) analysis was conducted on electron-transparent foils prepared through mechanical thinning to ~50 μm followed by twin-jet electropolishing. Mechanical evaluation was performed via uniaxial tensile testing at ambient temperature. To ensure statistical reliability, tests were conducted with three repetitions per condition. The testing protocol involved applying pre-strains of 2% and 10%, followed by artificial aging at 250 °C for 60 min to simulate long-term service conditions. Fracture resistance was assessed via Charpy V-notch impact testing (Zwick/Roell ZBC2452-B, ZwickRoell GmbH & Co. KG, Ulm, Germany). The N occurrence state was studied using an internal friction apparatus (MFP-1000, Institute of Metal Research, Chinese Academy of Sciences, Shenyang, China). 

## 3. Results and Discussions

### 3.1. Effect of Ti Addition on Microstructure and Mechanical Properties in High Residual N Steel

**Figure 2:**
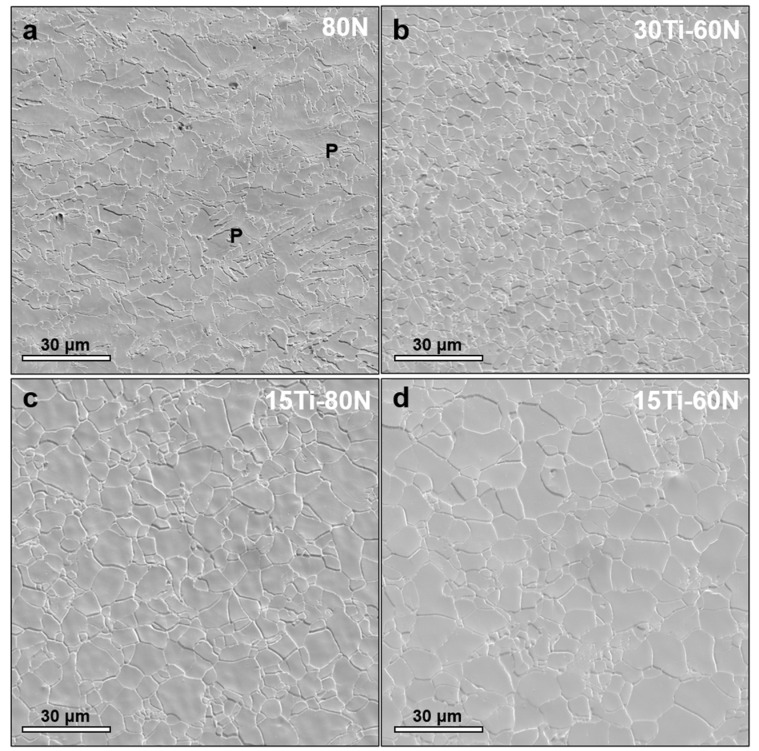
SEM images of experimental steels with varying N and Ti contents: (**a**) 80N, (**b**) 0.03Ti-60N, (**c**) 0.015Ti-80N, and (**d**) 0.015Ti-60N.

The microstructures of the experimental steels with varying Ti/N ratios were analyzed using scanning electron microscopy (SEM). Figure 2 presents the SEM images of the experimental steels. All the steels exhibited a microstructure consisting primarily of ferrite. The ferrite grains were found to be equiaxed and irregular in shape, with boundaries that were often curved and non-linear. Microstructural analysis confirmed that the 80N steel primarily consists of ferrite and small amount of pearlite, with no significant evidence of bainitic transformation under the given processing conditions (Figure 2c). In contrast, the Ti microalloyed steels mainly consisted of blocky ferrite grains, with only a small amount of pearlite (denoted as ‘P’ in Figure 2a). This change can be attributed to the addition of Ti, which promotes the formation of carbides and nitrides during solid-state precipitation upon cooling. During this process, large amounts of C and N are consumed from the solid solution. As C is a critical component in the formation of cementite (Fe_3_C), the reduction in C content significantly decreases cementite formation, thereby reducing the formation of pearlite.

Although the Ti/N ratio changes, the morphology of the ferrite remains relatively constant, while changes in grain size are more noticeable. The grain sizes of the 80N, 0.03Ti-60N, 0.015Ti-80N, and 0.015Ti-60N experimental steels were found to be 4.51 ± 0.23 μm, 4.83 ± 0.31 μm, 5.97 ± 0.35 μm, and 6.31 ± 0.28 μm, respectively. The 0.03Ti-60N composition, exceeding the stoichiometric Ti/N ratio of 3.42 [17], enabled secondary TiC precipitation. These nanoscale carbides exerted potent boundary pinning effects through Zener drag mechanisms, explaining the enhanced grain refinement beyond nitrogen’s solid solution effects. Conversely, sub-stoichiometric 0.015Ti variants lacked sufficient titanium for carbide formation, resulting in coarser microstructures with diminished precipitate-mediated grain boundary restraint.

**Figure 3:**
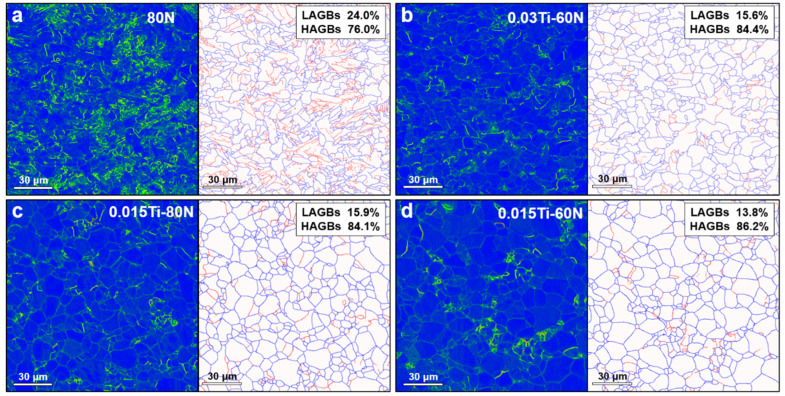
KAM and grain boundary images of experimental steels with varying N and Ti contents: (**a**) 80N, (**b**) 0.03Ti-60N, (**c**) 0.015Ti-80N, and (**d**) 0.015Ti-60N.

Figure 3 displays both the Kernel Average Misorientation (KAM) and grain boundary maps of the experimental steels. The KAM values for the 80N, 0.03Ti-60N, 0.015Ti-80N, and 0.015Ti-60N steels were 0.33°, 0.19°, 0.14°, and 0.14°, respectively. While the KAM values did not show a strong monotonic trend, the sample with the highest Ti content (0.03Ti-60N) exhibited a higher KAM value compared to the samples with lower Ti content (0.015Ti-80N and 0.015Ti-60N), suggesting that a higher density of fine precipitates (e.g., TiC in 0.03Ti-60N) may impede dislocation recovery, even as the overall dislocation density is reduced compared to the Ti-free steel. Compared to the Ti-free 80N steel, the Ti-containing steels exhibited lower KAM values, which can be attributed to the relatively lower dislocation density in the blocky ferrite microstructure. Regarding the grain boundary characteristics, boundaries were categorized based on their orientation difference. Low-angle grain boundaries (LAGBs) are defined by an orientation difference of 2°–15°, while high-angle grain boundaries (HAGBs) have an orientation difference greater than 15°. From Figure 3, it is evident that there is minimal variation in the fraction of HAGBs between the 0.03Ti-60N, 0.015Ti-80N, and 0.015Ti-60N steels. However, the 80N steel, which lacks Ti, shows the highest fraction of LAGBs. This is likely due to the absence of irregular ferrite or pearlite structures in the Ti-containing steels. The more regular, blocky ferrite morphology in these alloys tends to favor the formation of HAGBs over LAGBs.

**Figure 4:**
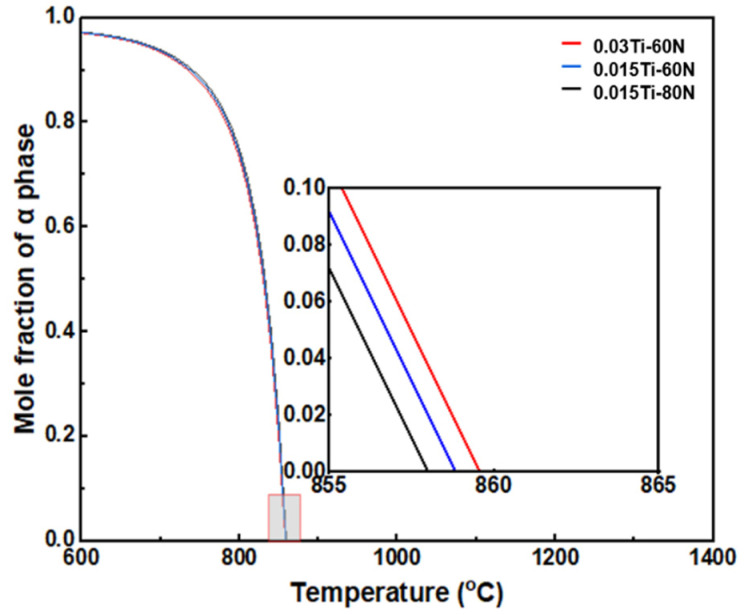
Evolution of the ferrite phase with temperature in experimental steels with varying Ti/N ratio.

To investigate the influence of Ti addition on the phase transformation process and its subsequent effect on the microstructure, the phase transition points of the experimental steels with varying Ti/N ratios were calculated through thermos-calc. The results are presented in Table 2 and Figure 4. Compared to the Ti-free experimental steels, the Ti-containing steels exhibited a decrease in both the austenite start temperature (Ac1) and the ferrite finish temperature (Ar3). Conversely, the austenite finish temperature (Ac3) and ferrite start temperature (Ar1) increased. These findings indicate that the addition of Ti expands the phase transformation region between austenite and ferrite. Furthermore, Ti effectively mitigates the influence of N on the phase transformation temperatures of the experimental steels. By fixing the N content, Ti raises the ferrite transformation temperature and reduces the undercooling temperature during the hot rolling process. These two combined effects lead to a microstructure predominantly consisting of blocky ferrite in the Ti-containing experimental steels. From a phase transformation perspective, this behavior promotes an increase in grain size, as the wider transformation zone encourages the formation of larger grains.

**Figure 5:**
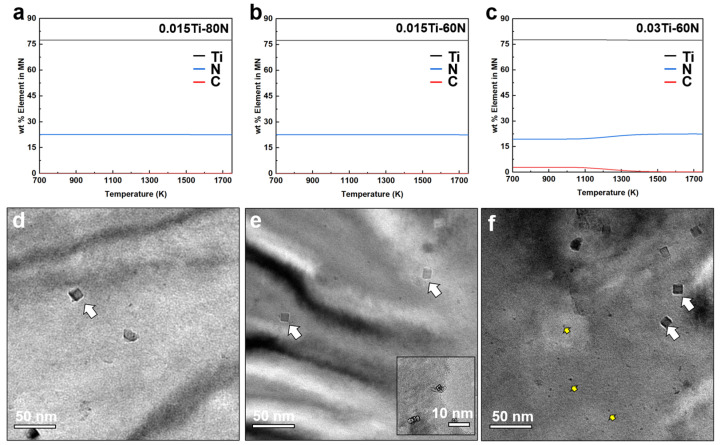
Evolution of the ferrite phase with temperature in experimental steels with different Ti/N contents. (**a**–**c**) Precipitation behavior of steels with varying Ti/N ratios. (**d**–**f**) Representative micrographs showing TiN precipitates in the three Ti-bearing steels.

Although the 0.03Ti-60N sample exhibits the highest Ar1 (Figure 4), it has the smallest grain size among the Ti-alloyed samples. This can likely be attributed to the pinning effect of the formed particles. Precipitation analysis for steels with different Ti/N ratios is shown in Figure 5a–c. The calculated MN-type precipitates (where M represents microalloying elements such as Ti and Nb, and N represents interstitial elements like C and N) are predominantly TiN for both the 0.015Ti-80N and 0.015Ti-60N samples. Additionally, the calculated liquidus and solidus temperatures for the 0.03Ti-60N, 0.015Ti-80N, and 0.015Ti-60N samples are presented in Table 3. The liquidus temperatures are 1498.6 °C, 1525.2 °C, and 1525.3 °C, respectively, while the corresponding solidus temperatures are 1525.1 °C, 1498.5 °C, and 1490.0 °C. As the total Ti plus N content increases, the liquidus temperature rises, and the solidus temperature decreases, which results in a broader solid–liquid interval. The predicted TiN precipitation temperatures for 0.03Ti-60N, 0.015Ti-80N, and 0.015Ti-60N are 1511.9 °C, 1505.3 °C, and 1504.8 °C, respectively. These results show a positive correlation between the TiN precipitation temperature and the combined Ti and N content.

In Figure 5d–f, representative micrographs of TiN in the three Ti-bearing steels are presented. Coarse, faceted TiN particles were detected in all samples, but their number density was very low, with sizes ranging from 50 to 100 nm. Interestingly, fine TiN precipitates, with sizes between 5 and 20 nm (inset of Figure 5e), were also observed. Given the low levels of Ti and N, and the fact that the computed TiN precipitation temperatures are only slightly above the solidus, it can be inferred that the 50–100 nm TiN particles are primarily liquid-borne precipitates, while the finer 5–20 nm TiN precipitates formed by solid-state precipitation during or after solidification. This interpretation aligns with previous studies, which report that liquid-precipitated TiN tends to be coarser and may impair ductility when present in high quantities [19,20,21,22,23]. In addition to TiN, sub-sized TiC precipitates (~5 nm) were occasionally observed in the 0.03Ti-60N sample. This observation aligns with thermodynamic predictions for 0.03Ti-60N (TiC present) and 0.015Ti-80N (TiC absent) shown in Figure 5a,c. However, for the 0.015Ti-60N sample (Figure 5e), sparse TiC particles were experimentally detected, even though the calculation predicted no TiC formation. The particle density in this alloy was much lower than in the 0.03Ti-60N sample. As predicted by the thermodynamic calculations in Figure 5a–c, the microscopy results broadly support the thermodynamic predictions, while the limited presence of TiC in the 0.015Ti-60N alloy suggests a narrow processing window that allows incipient TiC formation, which goes beyond the equilibrium prediction.

**Figure 6:**
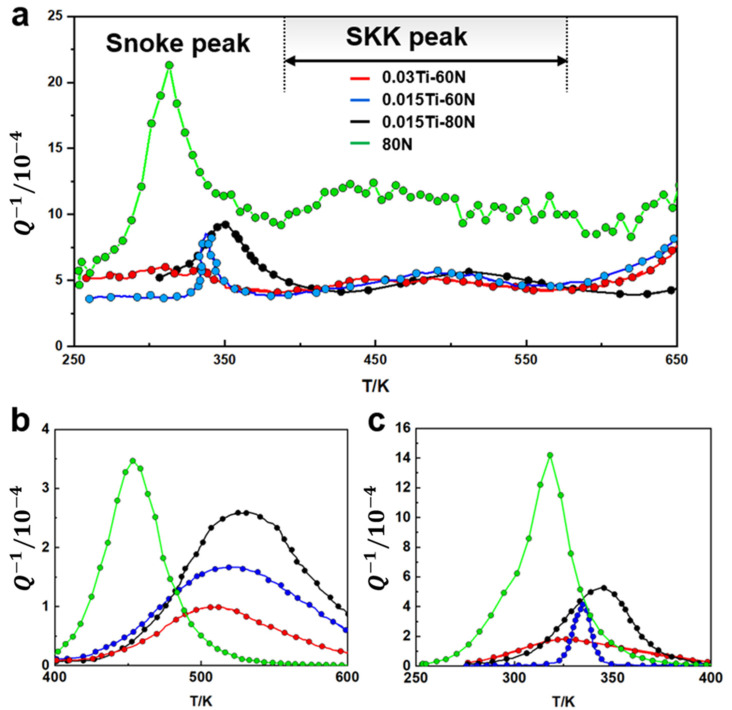
The internal friction experimental results for the experimental steels are presented as follows: (**a**) Original curve, (**b**) Snoek peak, and (**c**) SKK peak.

Internal friction spectroscopy was employed to investigate Ti’s influence on N occurrence states across systematically varied Ti/N stoichiometries. Characteristic damping peaks in the experimental spectra correspond to distinct interstitial relaxation mechanisms, the Snoek peak at approximately 340 K originates from stress-induced reorientation of interstitial atoms within body-centered cubic iron lattices, while the Snoek-Kê-Köster (SKK) peak near 520 K arises from interactions between dislocations and interstitial solutes [18]. These relaxation phenomena dissipate vibrational energy (a form of mechanical energy lost as heat during oscillatory stress) through atomistic processes including strain-induced solute ordering, dislocation kink migration, and grain boundary sliding mechanisms. Given the consistent carbon concentrations (0.04–0.06 wt%) across all specimens and analogous octahedral site occupation behaviors of C and N interstitials, variations in Snoek peak amplitude provide direct qualitative assessment of free N content [18]. Concurrently, SKK peak intensity exhibits dual dependence on mobile interstitial concentration and dislocation density, serving as an indicator of Cottrell atmosphere density—complexes where dislocations become pinned by interstitial solute clouds. Figure 6 demonstrates systematic attenuation of both relaxation signatures with Ti additions while their corresponding critical parameters and activation energies are summarized in Table 4 and Table 5, respectively. The 0.03Ti-60N steel exhibited a 28% lower Snoek amplitude than the 80N reference, indicating substantial reduction in free N. SKK intensity concurrently decreased by 34%, reflecting diminished dislocation–interstitial interactions. Both peaks progressively shifted toward lower temperatures (15–20 K reduction) with increasing Ti/N ratios, accompanied by 18–22% reductions in calculated activation energies. These phenomena collectively demonstrate Ti’s efficacy in immobilizing interstitial species through multiple mechanisms, preferential TiN nucleation consumes free nitrogen, substitutional-interstitial coupling reduces solute mobility, and precipitation-mediated dislocation pinning decreases dislocation density. The attenuated Cottrell atmosphere density evidenced by SKK suppression directly correlates with reduced strain aging susceptibility, which is a critical advancement for scrap-based EAF steels requiring precise interstitial management. Peak shifts further confirm Ti’s role in restricting interstitial mobility, with this stabilizing effect intensifying proportionally with Ti content elevation.

### 3.2. Effect of Ti Addition on Mechanical Properties and Strain Aging Behavior

**Figure 7:**
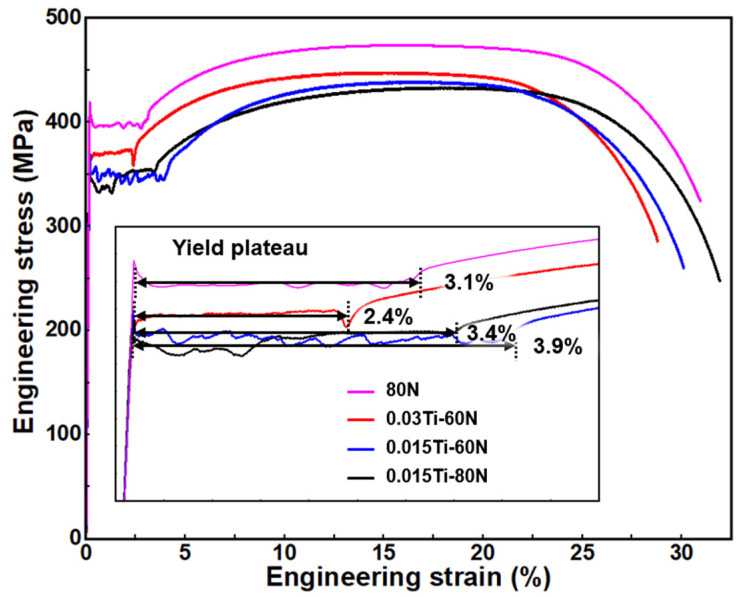
Engineering stress–strain curves of different experimental steels.

The effect of nitrogen occurrence states on the mechanical properties was evaluated from the tensile stress–strain curves (Figure 7) and the corresponding mechanical properties, including yield strength, ultimate tensile strength, and elongation (Table 6). As the total Ti and N content in the experimental steels increases, there is a general rise in upper yield strength, lower yield strength, ultimate tensile strength, and yield-to-tensile ratio. However, elongation tends to decrease gradually. Additionally, with an increase in the Ti/N ratio, the yield platform length becomes progressively shorter and displays oscillatory behavior. This phenomenon typically indicates the repeated pinning and depinning of dislocations by Cottrell atmospheres. Therefore, the shortened yield platform length is likely related to the reduction in interstitial atoms in the experimental steels. The three experimental steels with different Ti/N ratios mostly meet the required strength and plasticity of SAPH440 low-alloy steel. The 0.03Ti-60N experimental steel has slightly lower plasticity than the required 30%, while the 0.015Ti-60N and 0.015Ti-80N experimental steels exhibit tensile strengths slightly below the required 440 MPa. In contrast, the 80N sample shows higher strength than the standard requirement, which may be attributed to its irregular, elongated grain structure and reduced grain size.

**Figure 8:**
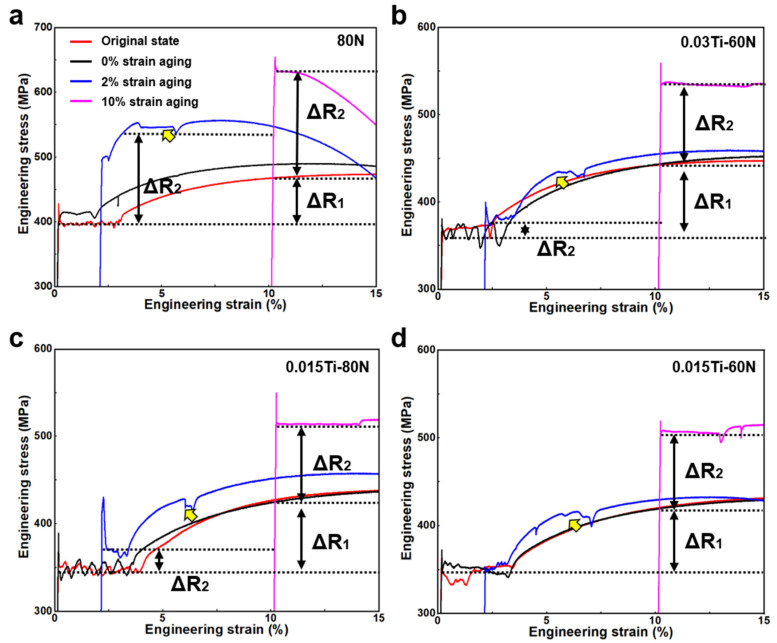
Engineering stress–strain curves of the experimental steels are presented as follows: Original curves and curves subjected to different strain aging conditions of 0% strain, 2% strain, and 10% strain for (**a**) 80N, (**b**) 0.03Ti-60N, (**c**) 0.015Ti-80N, and (**d**) 0.015Ti-60N, respectively. Yellow arrows indicate the positions of the secondary yielding points.

To simulate industrial processing conditions, including uncoiling (1–2% strain), leveling, and potential long-term storage, as well as end-user cold forming operations (5–10% strain), strain aging behavior was evaluated at 2% and 10% pre-strain levels. Artificial aging at 250 °C for 1 h accelerated natural aging equivalence to approximately one year of ambient exposure. Figure 8 presents tensile responses of 80N and Ti-microalloyed variants after pre-strain and aging treatments. Unprestrained steels exhibited negligible property changes post-aging except for the 80N reference. Following 2% pre-strain and aging, the 80N steel underwent profound mechanical transformation, in which the serrated yield plateau was replaced by a distinct two-stage yielding profile (indicated by yellow arrow, Figure 8a), signaling modified dislocation-solute interactions. This manifested as a dramatic yield strength elevation from 390 MPa to 537 MPa (+38%), coupled with critical ductility loss (15.8% vs. SAPH440’s ≥ 30% requirement). The strain aging sensitivity metric ΔR_2_ reached 147 MPa, predominantly attributable to Cottrell atmosphere formation between dislocations and mobile nitrogen interstitials.

Titanium microalloying fundamentally altered this response. At identical thermomechanical processing, as shown in Table 7, the 0.03Ti-60N and 0.015Ti-80N exhibited attenuated ΔR_2_ values near 30 MPa while 0.015Ti-60N demonstrated negligible ΔR_2_ (≈0 MPa). This progressive suppression of strain aging-induced hardening correlates with Ti’s efficacy in immobilizing N through precipitate formation and solute trapping. Although residual two-stage yielding persisted in Ti-modified steels, its diminished intensity reflects substantially reduced Cottrell atmosphere density. The 0.015Ti-60N composition, achieving near-complete strain aging resistance, validates stoichiometric optimization for interstitial control in scrap-based SAPH440 production.

Under 10% pre-strain and subsequent aging, all compositions exhibited a singular, continuous yield plateau (Figure 8), contrasting sharply with the serrated or multi-stage yielding observed at lower strains. This mechanical homogenization stems from extensive dislocation multiplication during severe plastic deformation, which simultaneously induces pronounced work hardening and fundamentally alters interstitial–dislocation interactions. Consequently, all steels manifested substantial strength elevation coupled with severe ductility reduction—consistent manifestations of pervasive strain aging. The underlying mechanism involves dislocation-mediated interstitial redistribution. During plastic deformation, crystalline defects propagate through the body-centered cubic lattice, generating high-density dislocation networks. Mobile N atoms rapidly diffuse toward tensile stress fields surrounding these dislocations, forming ordered Cottrell atmospheres that effectively pin dislocation motion. This dynamic explains the direct correlation between residual N content and strain aging intensity while higher N availability accelerates atmosphere formation, amplifying strength increases while degrading ductility. The Ti microalloyed steels demonstrated markedly attenuated property shifts despite identical thermomechanical processing. This suppression effect, quantitatively validated through internal friction spectroscopy, operates through Ti’s dual interstitial management mechanisms. Thermodynamic stabilization via TiN precipitation reduces free nitrogen concentration, and substitutional solute-dislocation interactions impede interstitial mobility. The resultant significant reduction in strain aging induced hardening confirms Ti’s efficacy in disrupting Cottrell atmosphere formation kinetics. Particularly in optimized Ti/N stoichiometries (0.015Ti-60N), near-complete mitigation of aging-induced embrittlement was achieved while maintaining SAPH440 mechanical specifications, which is a critical advancement for scrap-based structural steels requiring cold formability.

**Figure 9:**
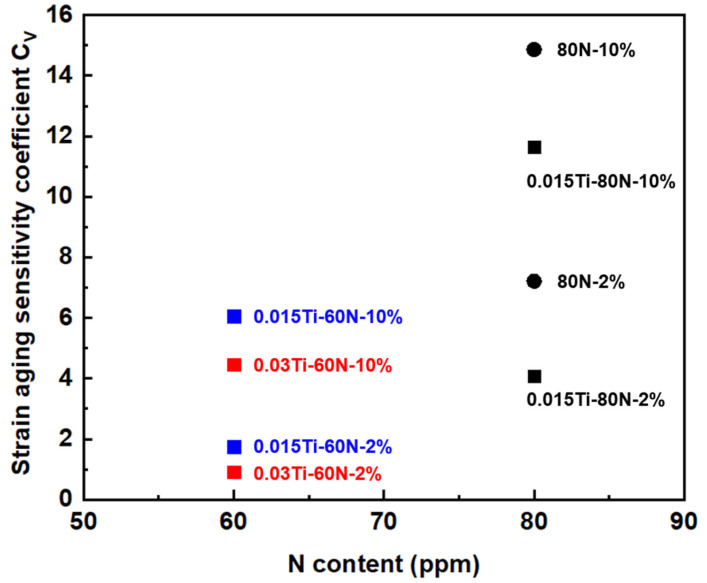
Effect of N and Ti on strain aging sensitivity coefficient C_V_ of experimental steels.

Impact energy measurements were conducted on the steels both before and after strain aging to assess the influence of changes in N occurrence states after strain aging (Table 8). Based on the impact energy, the strain-aging sensitivity coefficient (C_V_) was calculated by the following:

C_V_ = (A_k_ − A_ks_)/A_k_ (3)

where A_k_ represents the average impact absorption energy of the experimental steel before strain aging, and A_ks_ is the average impact absorption energy after the specified strain aging treatment, as per GB/T4160−2004 [24]. As shown in Figure 9, the addition of Ti significantly reduces the strain aging sensitivity coefficient of the experimental steels. For the 80 ppm N level, the C_V_ value reaches 7.43% for the 2% strain-aged sample. However, when Ti is added, the sensitivity coefficient decreases by 50%. Additionally, when comparing the C_V_ values of 15Ti-60N and 30Ti-60N, it is evident that as the Ti content increases, the strain aging sensitivity coefficient continues to decrease. This trend aligns with the internal friction test results, which indicate that Ti effectively stabilizes the free N atoms. These findings demonstrate the effective improvement of strain aging resistance in Ti-containing experimental steels. The addition of Ti helps reduce the strain aging sensitivity, with the sensitivity coefficient decreasing significantly as Ti content increases. This result highlights the critical role of Ti in enhancing the steel’s performance under strain aging conditions.

## 4. Conclusions

In summary, the present work investigates the influence of Ti microalloying on the N occurrence states and mechanical properties of experimental steels produced via the scrap-EAF process. The established TiN precipitation criterion based on the solubility product effectively guides Ti addition to suppress coarse liquid TiN inclusions while promoting fine solid-state TiN precipitates. Ti addition reduces free N atoms by 60–70% through efficient trapping mechanisms, which prevents the formation of Cottrell atmospheres and consequently leads to a dramatic reduction in strain aging sensitivity, with the strain aging index (ΔR_2_) decreasing from 147 MPa to nearly 0 MPa. This work presents an effective strategy for controlling nitrogen-induced strain aging in scrap-based EAF steels through tailored Ti microalloying, supported by thermodynamic calculations and experimental validation of both precipitation criteria and interstitial trapping mechanisms.

## Figures and Tables

**Table 1:** Experimental steel measured chemical composition (wt.%).

Sample	C	Si	Mn	Ti	N	Fe
80N	0.052	0.19	1.42	0	0.0079	Bal.
0.03Ti-60N	0.055	0.19	1.04	0.026	0.0065	Bal.
0.015Ti-80N	0.049	0.19	1.06	0.012	0.0081	Bal.
0.015Ti-60N	0.052	0.19	1.06	0.016	0.0062	Bal.

**Table 2:** Phase transformation temperature of experimental steels.

Sample	Ac1/℃	Ac3/℃	Ar1/℃	Ar3/℃
80N	654	842.5	842.5	654
0.03Ti-60N	532.7	859.6	859.6	532.7
0.015Ti-80N	526.8	858	858	526.8
0.015Ti-60N	521.2	858.8	858.8	521.2

**Table 3:** Transformation temperatures of experimental steels with varying Ti/N ratios.

Sample	Precipitation Point (°C)	Liquidus Point (°C)	Solidus Point (°C)
0.015Ti-80N	1505.3	1525.2	1498.5
0.015Ti-60N	1504.8	1525.3	1490
0.03Ti-60N	1511.9	1498.6	1525.1

**Table 4:** The parameters and activation energy of the Snoek peak in experimental steels with different Ti/N ratios.

Sample	Peak Value	Peak Temperature (K)	Frequency (Hz)	Activation Energy (eV)
80N	0.0142	338	2.388	0.8749
0.015Ti-80N	0.0052	346	2.453	0.8941
0.015Ti-60N	0.0043	336	2.574	0.8661
0.03Ti-60N	0.0018	325	2.552	0.837

**Table 5:** The parameters and activation energy of the SKK peak in experimental steels with different Ti/N ratios.

Sample	Peak Value	Peak Temperature (K)	Frequency (Hz)	Activation Energy (eV)
80N	0.0035	453	2.431	1.1815
0.015Ti-80N	0.0026	536	2.499	1.4045
0.015Ti-60N	0.0017	522	2.488	1.3668
0.03Ti-60N	0.001	513	2.431	1.3435

**Table 6:** Summary of mechanical properties of experimental steels.

Sample	YS/MPa	UTS/MPa	Elongation/%
80N	415 ± 1.7	490 ± 3.2	26.8 ± 0.8
0.03Ti-60N	374 ± 3.5	447 ± 4.9	28.8 ± 1.2
0.015Ti-80N	376 ± 2.8	438 ± 8	30.1 ± 0.9
0.015Ti-60N	364 ± 4.2	433 ± 6.1	31.9 ± 1.4

**Table 7:** Strength increments from work hardening (∆R1) and strain aging (∆R2) of the experimental steels under different pre-strain aging conditions.

Sample	∆R_1_/MPa (2%)	∆R_2_/MPa (2%)	∆R_1_/MPa (10%)	∆R_2_/MPa (10%)
80N	-	147 ± 2.5	78 ± 6.8	164 ± 7.2
0.03Ti-60N	-	35 ± 3.7	86 ± 7.1	88 ± 3.4
0.015Ti-80N	-	28 ± 2.1	88 ± 5.9	84 ± 2.8
0.015Ti-60N	-	0	89 ± 3.6	74 ± 4.3

**Table 8:** Impact energy and strain aging sensitivity coefficient of experimental steels before and after different strain aging conditions.

Sample	A_k_/J	A_ks_/J (2%)	A_ks_/J (10%)	C_V_ (2%)	C_V_ (10%)
80N	30.29	28.04	25.82	7.43	14.76
0.03Ti-60N	32.0	31.71	30.57	0.91	4.47
0.015Ti-80N	31.42	30.14	27.76	4.07	11.65
0.015Ti-60N	33.15	32.57	31.14	1.75	6.06

## Data Availability

The original contributions presented in this study are included in the article. Further inquiries can be directed to the corresponding author.
